# Acetaminophen induces accumulation of functional rat CYP3A via polyubiquitination dysfunction

**DOI:** 10.1038/srep21373

**Published:** 2016-02-22

**Authors:** Masataka Santoh, Seigo Sanoh, Masashi Takagi, Yoko Ejiri, Yaichiro Kotake, Shigeru Ohta

**Affiliations:** 1 Graduate School of Biomedical and Health Sciences, Hiroshima University, 1-2-3 Kasumi, Minami-ku, Hiroshima, 734-8553, Japan; 2 Molding Component Business Department, New business Development Division, Kuraray Co., Ltd. 1-1-3 Otemachi, Chiyoda-ku, Tokyo, 100-8115, Japan

## Abstract

Acetaminophen (APAP) is extensively used as an analgesic and antipyretic drug. APAP is partly metabolized to *N*-acetyl-*p*-benzoquinone imine, a reactive metabolite, by cytochrome P450 (CYP) 1A2, 2E1 and 3A4. Some reports have indicated that CYP3A protein production and its metabolic activity are induced by APAP in rats *in vivo*. The CYP3A subfamily is believed to be transcriptionally regulated by chemical compounds. However, the mechanism underlying these responses is not completely understood. To clarify these mechanisms, we assessed the effects of APAP on CYP3A1/23 protein levels according to mRNA synthesis and protein degradation in rat hepatocyte spheroids, a model of liver tissue, *in vivo*. APAP induced CYP3A1/23 protein levels and metabolic activity. However, no change in CYP3A1/23 mRNA levels was observed. Moreover, APAP prolonged the half-life of CYP3A1/23 protein. CYP3A is known to be degraded via the ubiquitin-proteasome system. APAP significantly was found to decrease levels of polyubiquitinated CYP3A1/23 and glycoprotein 78, an E3 ligase of CYP3A1/23. These findings demonstrate that APAP induces accumulation of functional CYP3A protein via inhibition of protein degradation. Our findings may lead to the determination of novel drug–drug interactions with APAP.

The cytochrome P450 (CYP) family comprises the major enzymes for drug and endogenous metabolism. CYP3A4, the predominant human CYP, is well known to contribute substantially to the metabolism of pharmaceutical agents^1^. From the perspective of drug–drug interactions (DDIs), the effects of chemical compounds on CYP3A protein expression need to be considered when evaluating drug metabolism and pharmacokinetics.

The CYP3A subfamily is believed to be transcriptionally regulated by chemical compounds via activation of the pregnane X receptor (PXR) and constitutive androstane receptor (CAR), both nuclear receptors. For instance, the antibiotic rifampicin is a potent inducer of human CYP3A through the activation of PXR^2^, and acetaminophen (APAP), analgesic and antipyretic drug induced CYP3A11 mRNA levels mediated by the activation of CAR in mice^3^. Moreover, Richert *et al.* reported that evaluation of mRNA levels was useful to detect the induction of CYP3A in rats and human hepatocytes^4^. However, Wolf *et al.* showed that APAP induced CYP3A protein levels in mice, although CYP3A11 mRNA levels were unchanged^5^. Based on these results, not only induction of mRNA levels but also protein stabilization needs to be considered when evaluating induction of CYP3A protein levels by chemical compounds. However, there are few reports regarding the induction of functional CYP3A via protein stabilization caused by inhibition of protein degradation.

Ubiquitin (Ub)-proteasome degradation (UPD) and autophagy-lysosomal degradation (ALD) comprise the typical protein degradation system. In UPD, the ubiquitination system first conjugates Ubs to proteins before proteasomes degrade the polyubiquitinated proteins. Damaged proteins and organelles resulting from cellular stress, such as nutrient starvation or oxidative stress, are degraded via lysosomes in ALD. Both UPD and ALD play roles in maintaining intracellular homeostasis by degrading damaged or excessive synthesized proteins^6^. CYP3A is localized to the endoplasmic reticulum (ER) membrane and predominantly undergoes ER-associated degradation (ERAD), which involves the Ub-proteasome system^7^.

In the present study, we examined the effects of APAP on CYP3A degradation using an *in vitro* system. Primary hepatocyte culture remains the gold standard *in vitro* model for metabolism studies; however, the activities of drug-metabolizing enzymes are rapidly lost during culture^8^. Several approaches have been developed for the maintenance of drug-metabolizing enzymes to overcome this limitation of hepatocyte culture. Three-dimensional culture models, such as spheroid culture models, gel-entrapment culture models and co-cultures of hepatic parenchymal with nonparenchymal cells, have been developed for the evaluation of drug metabolism^9^,^10^. These techniques have confirmed that CYP3A1/23 mRNA levels were maintained in hepatocyte spheroids formed by three-dimensional cell culture on microspace cell culture plates^11^. Moreover, CYP3A1/23 is the main CYP3A form in rat liver and has a high affinity for APAP metabolism^12^,^13^.

Consequently, we identified Ub-dependent proteasomal degradation dysfunction as the predominant mechanism underlying the induction of CYP3A1/23 protein levels and metabolic activity in response to APAP exposure in a rat hepatocyte spheroids culture model. These findings may represent a novel mechanism of APAP-induced hepatotoxicity through the metabolic activation by CYP3A and may lead to the determination of novel CYP3A DDI.

## Results

### CYP3A1/23 protein levels in hepatocyte spheroids during culture

**Figure 1:**
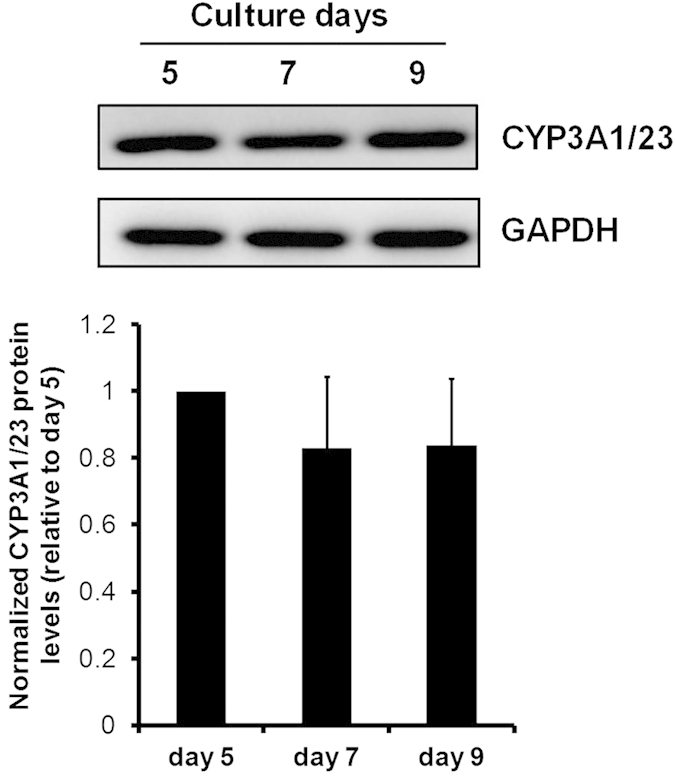
CYP3A1/23 protein levels in hepatocyte spheroids during culture. Rat hepatocyte spheroids were harvested at days 5, 7, and 9. Representative results of CYP3A1/23 and GAPDH immunoblotting analyses of cell lysates (5 μg of protein) are shown in the top panel. GAPDH was measured as a loading control. Cropped blots were shown and the full length-blots were presented in Supplementary Fig. 1. The results of densitometric quantification of CYP3A1/23 protein levels are shown in the bottom panel. Results are expressed as means ± S.D. (*n* = 3 independent experiments).

Spheroid formation was achieved by the inoculation of hepatocytes for 5 days. We evaluated CYP3A1/23 protein levels in hepatocyte spheroids at day 5. Figure 1 shows CYP3A1/23 protein levels in hepatocyte spheroids between days 5 and 9. CYP3A1/23 protein levels were maintained stably by achievement of spheroid formation. Therefore, hepatocyte spheroids (day 5) were used in all subsequent experiments.

### Effects of APAP on CYP3A1/23 mRNA levels and protein levels and activity

**Figure 2:**
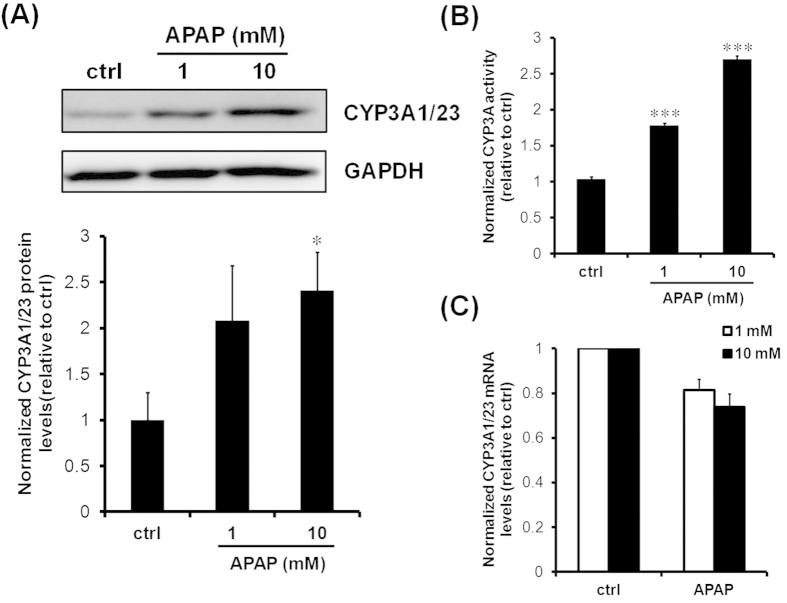
Effects of APAP on CYP3A1/23 protein levels and activities and mRNA levels. Rat hepatocyte spheroids (day 5) were exposed to APAP (1 and 10 mM) for 24 h. (**A**) Representative results of CYP3A1/23 and GAPDH immunoblotting analyses of cell lysates (5 μg of protein) are shown in the top panel. GAPDH was measured as a loading control. Cropped blots were shown and the full-length blots were presented in Supplementary Fig. 1. The results of densitometric quantification of CYP3A1/23 protein levels are shown in the bottom panel. Results are expressed as means ± S.D. (*n* = 3 independent experiments, *P < 0.05 vs. ctrl, Tukey’s test). (**B**) CYP3A metabolic activity was measured with luciferin-IPA. CYP3A metabolic activity was normalized to protein levels. Results are expressed as means ± S.D. (*n* = 3 independent experiments, ***P < 0.001 vs. ctrl, Tukey’s test). (**C**) CYP3A1/23 mRNA levels were measured by qRT-PCR analyses. CYP3A1/23 mRNA levels were normalized to β-2-microglobulin (β-2-m) mRNA levels. Results are expressed as means ± S.D. (*n* = 3 independent experiments).

**Figure 3:**
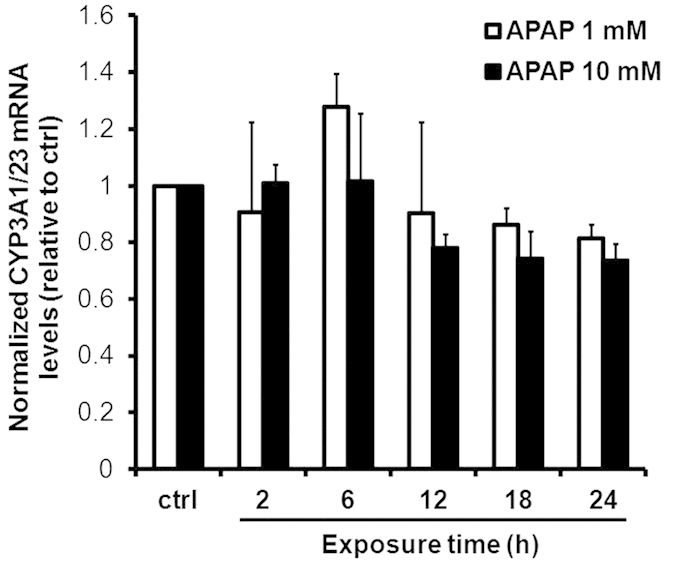
Effects of APAP on CYP3A1/23 mRNA levels over time. Rat hepatocyte spheroids (day 5) were exposed to APAP (1 and 10 mM) for 2, 6, 12, 18 and 24 h before harvesting. CYP3A1/23 mRNA levels were measured by qRT-PCR analyses and normalized to β-2-m mRNA levels. Results are expressed as means ± S.D. (*n* = 3 independent experiments).

Figure 2A,B show changes in CYP3A1/23 protein levels according to immunoblotting analyses and CYP3A activity according to luciferin-IPA probes in rat hepatocyte spheroids after 24 h exposure to 1 and 10 mM APAP, respectively. Both CYP3A1/23 protein levels and CYP3A metabolic activity significantly increased by 1.5- to 2.5-fold compared with controls after exposure to 1 and 10 mM APAP, respectively. However, induction of CYP3A1/23 mRNA levels in response to changes in CYP3A1/23 protein levels and metabolic activity was not observed. Rather, CYP3A1/23 mRNA levels tended to decline after exposure to 1 and 10 mM APAP (Fig. 2C). To confirm the time dependency of CYP3A1/23 mRNA levels in spheroids after exposure to APAP, we examined CYP3A1/23 mRNA levels at several time points after exposure to APAP. The results in Fig. 3 demonstrate that the induction of CYP3A1/23 mRNA levels was not observed at any time point, although mRNA levels slightly declined between 12 and 24 h after exposure to 10 mM APAP. Therefore, the induction of CYP3A1/23 protein contents and metabolic activity may be attributable to CYP3A1/23 protein stabilization in response to APAP.

### Degradation of CYP3A1/23 protein levels and inhibition of protein degradation in response to APAP

**Figure 4:**
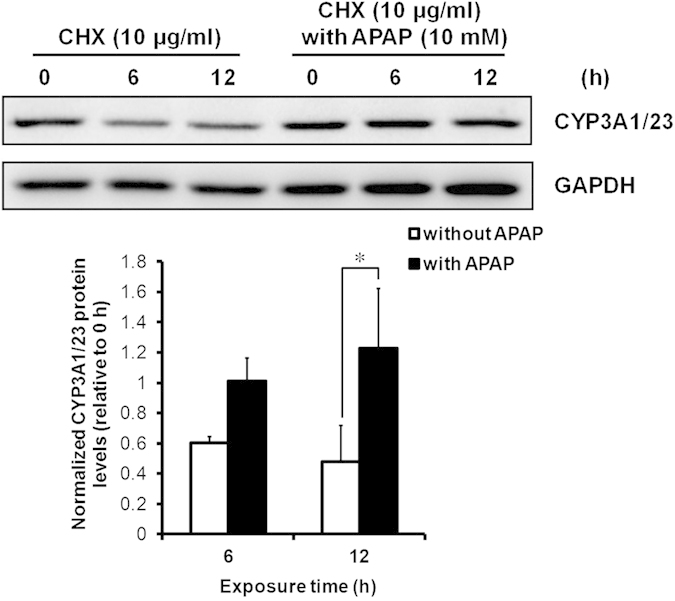
Effects of APAP on CYP3A1/23 protein degradation. Rat hepatocyte spheroids (day 5) were exposed to cycloheximide (10 μg/ml) with or without APAP (10 mM) for 6 or 12 h before harvesting. Representative results of CYP3A1/23 and GAPDH immunoblotting analyses of cell lysates (5 μg of protein) are shown in the top panel. GAPDH was measured as a loading control. Cropped blots were shown and the full-length blots were presented in Supplementary Fig. 1. The results of densitometric quantification of CYP3A1/23 protein levels are shown in the bottom panel. Results are expressed as means ± S.D. (*n* = 3 independent experiments, *P < 0.05 vs. without APAP, Tukey’s test).

We examined the effects of APAP on the degradation of CYP3A1/23 protein levels in rat hepatocyte spheroids. Immunoblotting analyses with lysates from rat hepatocyte spheroids exposed to 10 μg/ml cycloheximide, a potent inhibitor of protein synthesis, indicated the degradation of CYP3A1/23 protein after 6-h exposure. In contrast, co-exposure to cycloheximide and 10 mM APAP did not result in the degradation of CYP3A1/23 protein contents until 12 h after exposure (Fig. 4). These results indicate that APAP may inhibit the degradation of CYP3A1/23 protein levels.

### Effects of APAP on proteasome activities and Nrf2 protein levels

**Figure 5:**
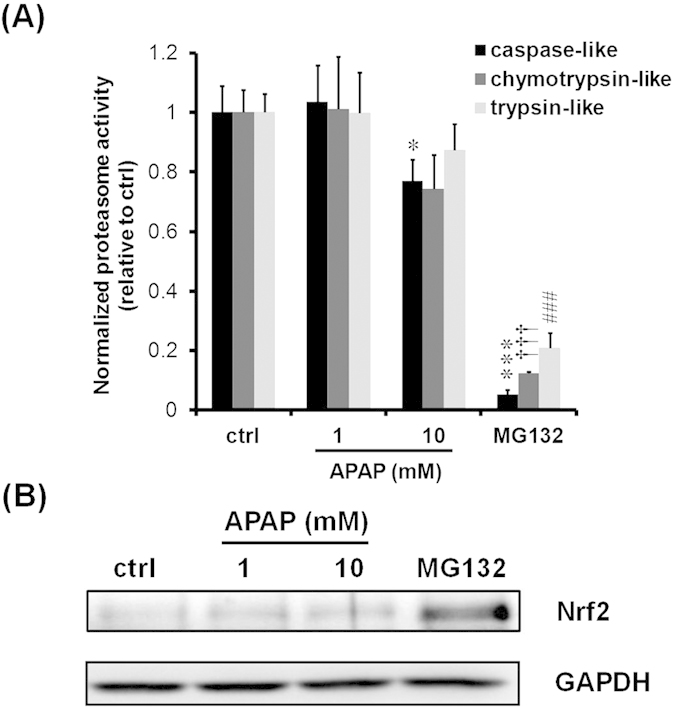
Effects of APAP on proteasome activity and Nrf2 protein levels. Rat hepatocyte spheroids (day 5) were exposed to MG132 (10 μM, 6 h) and APAP (1 and 10 mM, 24 h) and harvested. (**A**) Proteasome activities were measured with three proteasome substrates and normalized to protein contents. Results are expressed as means ± S.D. (*n* = 3 independent experiments, *P < 0.05, ***P < 0.001, ^†††^*P* < 0.001, ^###^*P* < 0.001 vs. ctrl, Tukey’s test). (**B**) Representative results of Nrf2 and GAPDH immunoblotting analyses of cell lysates (10 μg of protein) are shown. GAPDH was measured as a loading control. Cropped blots were shown and the full-length blots were presented in Supplementary Fig. 1.

To investigate the predominant target of CYP3A1/23 degradation inhibition in response to APAP, we evaluated the effects of APAP on proteasome activity. Three different artificial proteasome substrates were used to examine caspase-, chymotrypsin-, and trypsin-like proteasomal activities. We confirmed that exposure to 10 μM MG132, a potent proteasome inhibitor, reduced all proteasome activities in spheroids. However, 1 mM APAP had no effect on proteasome activities, although 10 mM APAP decreased caspase- and chymotrypsin-like activities (Fig. 5A). To examine whether reductions in proteasome activities contributed to the induction of CYP3A1/23 protein, NF-E2-related factor 2 (Nrf2) protein, another substrate of the Ub-proteasome system, was measured^14^. The accumulation of Nrf2 proteins was induced by exposure to 10 μM MG132 for 6 h. However, both 1 and 10 mM APAP had no effect on Nrf2 protein levels (Fig. 5B). These results indicate the predominant target of CYP3A degradation inhibition in response to APAP is not the proteasome complex.

### Effects of APAP on the CYP3A ligases, gp78 and CHIP

**Figure 6:**
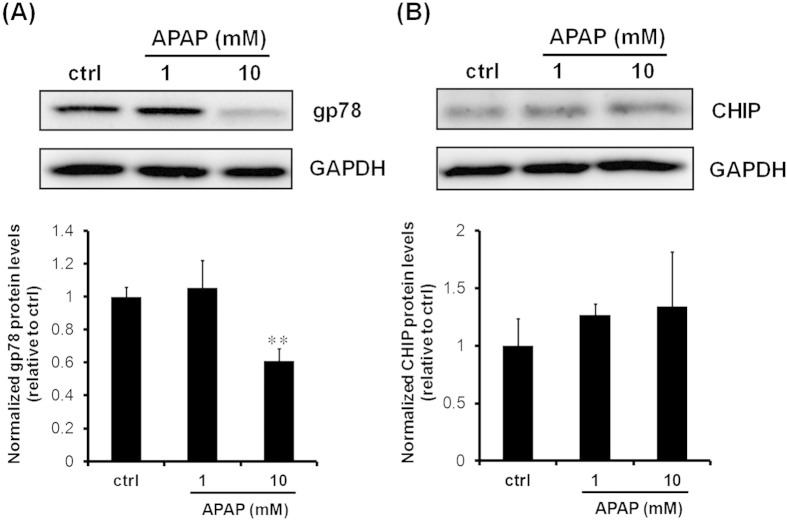
Effects of APAP on gp78 and CHIP protein levels. Rat hepatocyte spheroids (day 5) were exposed to APAP (1 and 10 mM) for 24 h before harvesting. (**A**) Representative results of gp78 and GAPDH immunoblotting analyses of cell lysates (10 μg of protein) are shown in the top panel. GAPDH was measured as a loading control. Cropped blots were shown and the full-length blots were presented in Supplementary Fig. 1. The results of densitometric quantification of gp78 protein levels are shown in the bottom panel. Results are expressed as means ± S.D. (*n* = 3 independent experiments, **P < 0.01 vs. ctrl, Tukey’s test). (**B**) Representative results of CHIP and GAPDH immunoblotting analyses of cell lysates (10 μg of protein) are shown in the top panel. Cropped blots were shown and the full-length blots were presented in Supplementary Fig. 1. The results of densitometric quantification of CHIP protein levels are shown in the bottom panel. Results are expressed as means ± S.D. (*n* = 3 independent experiments).

**Figure 7:**
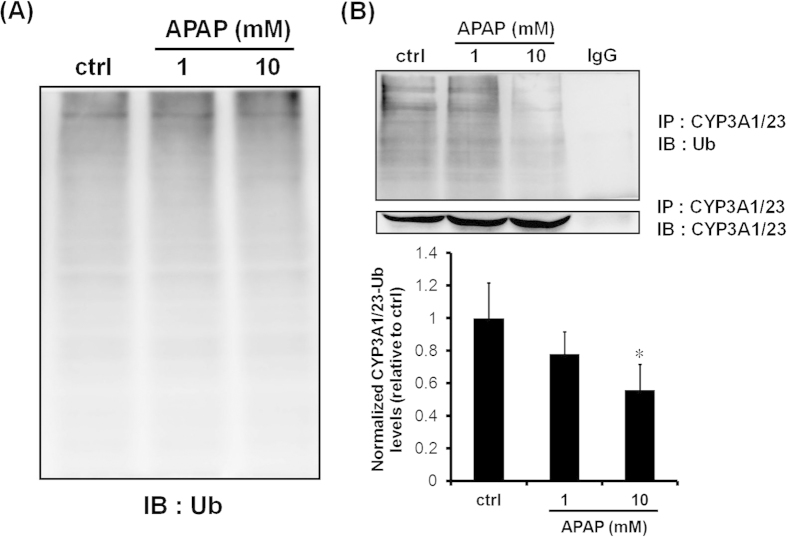
Effects of APAP on ubiquitination levels of CYP3A1/23 protein. (**A**) Rat hepatocyte spheroids (day 5) were exposed to APAP (1 and 10 mM) for 24 h before harvesting. A representative result of ubiquitin immunoblotting analyses of cell lysates (10 μg of protein) is shown. (**B**) Rat hepatocyte spheroids (day 5) were exposed to APAP (1 and 10 mM, 24 h) with MG132 (10 μM, 6 h) before harvesting. Representative results of ubiquitin and CYP3A1/23 immunoblotting analyses of CYP3A1/23 immunoprecipitates are shown in the top panel. Cropped blots were shown and the full-length blots were presented in Supplementary Fig. 1. The results of densitometric quantification of CYP3A1/23-ubiquitin levels are shown in the bottom panel. Results are expressed as means ± S.D. (*n* = 3 independent experiments, *P < 0.05, vs. ctrl, Tukey’s test).

We hypothesized that APAP decreases the protein levels of glycoprotein 78 (gp78) or C terminus of Hsp70-interacting protein (CHIP), known to be E3 Ub ligases of the CYP3A protein. A reduction in the levels of gp78 protein was observed by immunoblotting analyses using lysates from rat hepatocyte spheroids exposed to 10 mM APAP for 24 h. However, only a slight induction in CHIP protein levels was observed in spheroids exposed to 10 mM APAP for 24 h (Fig. 6A,B). Significant reductions in gp78 protein levels may lead to decreased polyubiquitination of CYP3A1/23 protein related to the protein degradation process. Therefore, we conducted Ub immunoblotting analyses of CYP3A1/23 immunoprecipitates prepared from cell lysates. First, we confirmed that APAP had no effect on total ubiquitination of proteins (Fig. 7A). The results shown in Fig. 7B demonstrated that APAP reduced the polyubiquitination of CYP3A1/23 protein levels (Fig. 7B). These findings indicate that inhibition of the degradation of CYP3A1/23 protein by APAP may be due to decreased ubiquitination of CYP3A1/23 protein.

## Discussion

In the present study, we found that APAP increased rat CYP3A1/23 protein levels and the metabolic activity of CYP3A-specific substrates via inhibition of protein degradation associated with reductions in gp78 protein levels.

The transcriptional induction of functional CYP3A is reportedly mediated by the activation of nuclear receptors in response to exposure to several drugs such as dexamethasone (Dex), phenobarbital and rifampicin^2^. In the present study, both rat CYP3A1/23 protein levels and CYP3A metabolic activity were significantly increased compared with controls after exposure to 1 and 10 mM APAP in rat hepatocyte spheroids. However, the induction of CYP3A1/23 mRNA levels was not found to be proportional to the observed increases in CYP3A1/23 protein expression and CYP3A metabolic activity. Sprague-Dawley rat liver expressed CYP3A1/23, CYP3A2, CYP3A9, and CYP3A18^12^,^15^,^16^,^17^. The anti-CYP3A1 antibody used in this study could cross-react with other CYP3A isoforms due to similarity of amino acid sequences. However, a previous report indicated that Dex induced CYP3A1/23 mRNA levels, whereas CYP3A2, CYP3A9, and CYP3A18 mRNA levels were not affected by Dex in cultured rat primary hepatocytes^18^. Therefore we evaluated the specificity of anti-CYP3A1 IgG through the immunoblotting results using cell lysates from Dex-induced rat hepatocyte spheroids. The immunoblotting results showed that APAP (1 and 10 mM) and Dex enhanced the intensity of bands recognized by anti-CYP3A1 IgG (see Supplementary Fig. S2). From these results we concluded that the detectable bands recognized by anti-CYP3A1 IgG are CYP3A1/23. Therefore, CYP3A1/23 protein stabilization, rather than transcriptional activation, is likely to underlie the effects of APAP. There are a few reports of other chemicals changing levels through the stimulation or inhibition of CYP3A protein degradation^19^,^20^. 3,5-Dicarbethoxy-2,6-dimethyl-4-ethyl-1,4-dihydropyridine, a structural analog of the dihydropyridine Ca^2+^ antagonists, inactivates CYP3A through the heme-modification and rapid degradation of CYP3A protein^19^,^21^. The macrolide antibiotic, triacetyloleandomycin (TAO), increases CYP3A protein levels through inhibition of degradation^20^. Faouzi *et al.* posited that TAO-complexation stabilizes CYP3A protein and render it less susceptible to ubiquitination, possibly by concealing the target for Lys-residues required for polyubiquitination^21^. However, CYP3A protein accumulated in response to TAO could not have metabolic activity as TAO is a specific CYP3A mechanism-based inhibitor through the chemical modification of the heme subunit of the CYP3A4 protein^21^. With regard to APAP, previous report indicated that CYP3A4 protein is stabilized, and its activity is induced by APAP in a HepG2 cell line stably expressing CYP3A4, but not identified the mechanism underlying the inhibition of CYP3A degradation^22^. Our results corroborate this report (Figs 2 and 4). Moreover, we examined the effects of APAP on the CYP3A stability and its mechanism using rat hepatocyte spheroids, which are expected to maintain *in vivo* liver function and the cellular environment, whereas the above report used CYP3A4-expressing cells. Hepatocyte spheroids were formed from day 5 after the seeding of rat hepatocytes onto microspace cell culture plates^11^. Regularly sized spheroids can be formed on the plate as the bottom surface of each well consists of regularly spaced square compartments (200-μm length × 200-μm width × 50-μm depth). Protein expression levels of CYP3A1/23, the major CYP3A isoform in rat liver^12^, were maintained to days 7 and 9 (Fig. 1).

Zhang *et al.* reported that APAP induces mouse CYP3A11 mRNA levels via CAR activation^3^. In the present study, APAP decreased rat CYP3A1/23 mRNA levels by 80% compared with control values (Figs 2C and 3). A species difference in the required APAP concentrations for CAR transactivation between mice and rats may underlie this discrepancy. The reductions in CYP3A1/23 mRNA levels in response to APAP exposure were not reflected by the increase in CYP3A1/23 protein levels (Fig. 2A,C). The accumulation of CYP3A1/23 protein may partly have contributed to the suppression of CYP3A1/23 mRNA levels through a negative feedback mechanism.

In rat hepatocyte spheroids treated with cycloheximide, an inhibitor of protein synthesis, the half-life of CYP3A1/23 protein was found to be approximately 11 h (Fig. 4). A previous study reported that the half-life for the degradation of CYP3A was approximately 9 h in rat cultured hepatocytes, in general agreement with our results^20^. The CYP family demonstrates a range of half-lives for degradation. CYP3A reportedly has a short half-life, whereas native CYP2B1 and CYP2C11 exhibit long half-lives (>20 h), and native CYP2E1 has biphasic half-lives of 7 and 38 h^7^. Although CYP3A is degraded by the Ub-dependent proteasomal system, native CYP2B1 and CYP2C11 undergo ALD, a process associated with long half-lives^7^,^23^,^24^. Therefore, APAP may induce CYP2E1 protein levels via protein stabilization as CYP2E1 is also degraded by not only ALD but also UPD as with CYP3A4^25^.

APAP at a concentration of 10 mM decreased caspase-like and chymotrypsin-like proteasomal activities (Fig. 5A). Previous reports indicated that one of the hepatic protein-binding targets of APAP is a proteasome subunit alpha type 3 in mouse^26^. Therefore, APAP inhibition of proteasome activity may be due to alteration of 20S proteasome structures. However, as shown in Fig. 5B, there was no induction of Nrf2 protein, which is degraded by Ub proteasome system^14^, in response to APAP. These results indicate reduction in proteasome activity had no effect on the inhibition of CYP3A degradation by APAP.

Recently, the pathways of CYP degradation have been revealed. CYP3A undergoes UPD in the ERAD, which involves in the phosphorylation, ubiquitination and extraction of CYP3A from ER to cytoplasm prior to degradation by the 26S proteasome^27^,^28^,^29^. Ubiquitination processes are associated with gp78 and CHIP as the E3-Ub ligases and UBC7 and UbcH5a as E2 Ub-conjugating enzymes^28^,^30^. Gp78 was identified as a receptor for tumour cell autocrine motility factor, which facilitates tumour metastasis^31^. Moreover, another report indicated that gp78 is a RING finger-dependent Ub ligase that plays a role in ERAD^32^. In contrast, CHIP reportedly functions as an E3 ligase in combination with heat shock protein 70 (HSP70) and HSP90^33^,^34^,^35^. In the present study, 10 mM APAP decreased polyubiquitinated CYP3A1/23 protein levels (Fig. 7B) but did not alter the total Ub levels of proteins (Fig. 7A). Furthermore, 10 mM APAP significantly reduced gp78 protein levels and slightly induced CHIP protein levels (Fig. 6). Kim *et al.* reported that gp78 knockdown induced functional CYP3A without affecting CHIP protein levels in rat primary hepatocytes treated with Dex^28^. These reports corresponded to the induction of functional CYP3A via the reduction in gp78 protein levels caused by 10 mM APAP in this study. A previous *in vitro* study demonstrated that gp78 alone specifically mediated the subsequent ubiquitination of ubiquitin-fused GST, but not intact GST^36^. A further report suggested gp78 may play a role in polyubiquitination, whereas CHIP may initially ubiquitinate proteins^28^. Therefore, the results of the present study demonstrate that the polyubiquitination of CYP3A1/23 levels is dependent on gp78 protein levels. However, 1 mM APAP had no effect on gp78 protein levels despite the induction of CYP3A1/23 protein levels and metabolic activity (Figs 2A,B and 6A). 1 mM APAP prolonged CYP3A1/23 protein levels in cycloheximide-chase assay as well as 10 mM APAP (see Supplementary Fig. S3). Therefore other factors may reduce the activity of ubiquitination as the level of polyubiquitinated CYP3A1/23 protein was reduced without the reduction of gp78 protein levels by 1 mM APAP (Fig. 7B).

In summary, the findings of the present study suggested APAP increased CYP3A1/23 protein levels through the reduction of gp78 protein levels and the inhibition of CYP3A1/23 protein degradation. Furthermore, accumulated CYP3A in response to APAP was shown to be functional as significant metabolic activity was detected in spheroids. APAP is partly metabolized to *N*-acetyl-*p*-benzoquinone imine (NAPQI) by CYP1A2, 2E1 and 3A4^37^. As NAPQI is a toxic reactive metabolite, the metabolic activation of CYPs should be considered when administering APAP. Stabilization of CYP3A protein induced by APAP may contribute to the formation of NAPQI by CYP3A and subsequent hepatotoxicity. It is important to consider the DDI of APAP with other CYP3A-mediated drugs as APAP is a widely used analgesic and antipyretic drug, and CYP3A has a critical role in the metabolism of pharmaceutical agents. The evaluation of structure–activity relationships based on the chemical structure of APAP may identify other pharmaceutical agents that cause the accumulation of functional CYP3A. Further examination of the species differences observed in the findings of the present is required. The findings of the present study may lead to the determination of novel DDIs with APAP.

## Methods

### Materials

Dulbecco’s modified Eagle’s medium (DMEM)/Nutrient Mixture F-12 Ham, APAP, l-glutamine, l-ascorbic acid, anti-GAPDH antibody and Triton^TM^ X-100 were purchased from Sigma-Aldrich (St. Louis, MO, USA). Collagenase, insulin human recombinant and cycloheximide were purchased from Wako Pure Chemical Industries (Osaka, Japan). Soybean trypsin inhibitor powder, HEPES buffer (1 M) and Dynabeads^®^ Protein G were purchased from Thermo Fisher Scientific (Gibco^®^; Waltham, MA, USA). 2-Mercaptoethanol, dexamethasone, Nonidet^®^ P-40 and polyoxyethylene sorbitan monolaurate were purchased from Nacalai tesque (Kyoto, Japan). MG132 (Z-Leu-Leu-Leu-H aldehyde) was purchased from Peptide Institute (Osaka, Japan). Proteasome substrates II, III and VI, and Fluorogenic and anti-rabbit CYP3A1 antibodies were purchased from Merck Millipore (Calbiochem^®^; Darmstadt, Germany). Penicillin G potassium and streptomycin were purchased from Meiji Seika Pharma (Tokyo, Japan). Anti-mouse CYP3A1, anti-rabbit CHIP, anti-rabbit gp78-2, anti-rabbit Nrf2 and anti-mouse Ub antibodies were purchased from Santacruz Biotechnology (Dallas, Texas, USA). P450-Glo^TM^ CYP3A assay with luciferin-IPA was purchased from Promega (Fitchburg, WI, USA).

### Animals

Male Crj:CD (SD) rats (7 weeks old) were purchased from Charles River Laboratories, Japan. All animal protocols were approved by the animal ethics committee of Hiroshima University and all experimental procedures were performed in accordance with the guidelines of the animal ethics committee of Hiroshima University.

### Hepatocyte isolation and cell culture

Primary rat hepatocytes were isolated from SD rats using a collagenase liver perfusion method as previously described^38^. Hepatocytes (2.5 × 10^5^) were seeded onto 24-well microspace culture plates coated with 0.01% poly-l-lysine (Elplasia^®^, Kuraray Co., Ltd.). Cells were cultured with supplemented DMEM in an incubator (5% CO_2_ and 95% O_2_ at 37 °C). Culture medium contained 10% foetal bovine serum, 2 mM l-glutamine, 100 units/ml penicillin, 100 μg/ml streptomycin, 10 mM nicotinamide, 50 μM 2-mercaptoethanol, 100 nM dexamethasone, 520 μM l-ascorbic acid, 1 μg/ml insulin and 5 mM HEPES.

### Exposure of hepatocyte spheroids to APAP

APAP (1 and 10 mM) were dissolved in cultured medium directly.

### Immunoblotting analysis

Cells were harvested in lysis buffer consisting of 25 mM Tris-HCl (pH 7.6), 150 mM NaCl, 1% Nonidet^®^ P-40, 1% sodium deoxycholate, 0.1% SDS, protease inhibitor cocktail, 1 mM NaF and 1 mM Na_3_VO_4_. When evaluating the ubiquitination of proteins, 10 mM *N*-ethylmaleimide was added. Lysate supernatants were subjected to 10% SDS-PAGE followed by electroblotting onto a PVDF membrane. Because previous report suggested that CYP3A1 and CYP3A23 is the same form^15^, CYP3A1/23, GAPDH, gp78-2, CHIP and Nrf2 were immunoblotted with primary rabbit polyclonal antibodies (AB1253, G9545, sc-33541, sc-66830 and sc-13032, respectively). Ub was immunoblotted with primary mouse monoclonal antibody (sc-8017). Goat anti-rabbit HRP (A9169) and rabbit anti-mouse HRP (A9044) were used as the secondary antibody. Approximately 5% skimmed milk in Tween TBS was used for blocking and for dilutions of all primary and secondary antibodies. Densitometric quantification was performed using ImageQuant LAS 4000 mini (GE Healthcare, Chalfont St. Giles, Buckinghamshire, UK).

### qRT-PCR analysis

Total RNA was extracted using an SV Total RNA Isolation System (Promega, Fitchburg, WI). Reverse transcription to cDNA was performed using AMV Reverse Transcription Kits (Promega) and oligo dT primers. Quantitative real-time PCR was performed on a Bio-Rad CFX system using SYBR^®^ Green PCR kits. The expression levels of target mRNAs were normalized to the expression of β-2-m. Gene expression levels were calculated using the standard curve method. Standard curve samples were produced by QIAquick^®^ Gel Extraction kits (QIAGEN, Hilden, Germany). The following primers were used in the present study: CYP3A1/23 forward: 5′-GAAACTGCAGGAGGAGATCG-3′; CYP3A1/23 reverse: 5′-TCACAGTATCATAGGTGGGAG-GT-3′; β-2-m forward: 5′-CGAGACCGATGTATATGCTTGC-3′; β-2-m reverse: 5′-GTCCAGA-TGATTCAGAGCTCCA-3′.

### CYP3A1/23 immunoprecipitation

Cells were harvested in lysis buffer consisting of 10 mM Tris-HCl (pH 7.6), 150 mM NaCl, 1% Triton^TM^ X-100, 1% sodium deoxycholate, 1 mM EDTA and 20% glycerol, protease inhibitor cocktail, 1 mM NaF, 1 mM Na_3_VO_4_ and 10 mM *N*-ethylmaleimide. For the immunoprecipitation of CYP3A1/23 protein, 400 μg of cell lysate was immunoprecipitated with anti-rabbit polyclonal CYP3A1 antibody and Dynabeads^®^ Protein G. Immunoprecipitated CYP3A1/23 was immunoblotted with anti-mouse monoclonal CYP3A1 antibody and anti-mouse monoclonal Ub antibody (sc-53246 and sc-8017).

### CYP3A activity

CYP3A activity was measured using a P450-Glo^TM^ CYP3A assay with luciferin-IPA. Hepatocytes were washed twice with PBS (−) and incubated with 3 μM luciferin-IPA in DMEM without phenol red for 30 min under 5% CO_2_ and 95% O_2_ at 37 °C. The formations of luciferin, a luciferin-IPA metabolite, after incubation in detection buffer for 20 min at room temperature were measured as luminescence by Ensipire^TM^ (PerkinElmer, Waltham, MA, USA).

### Proteasome activity

Cells were harvested in lysis buffer consisting of 50 mM HEPES, 150 mM NaCl, 1.5 mM MgCl_2_, 1 mM EGTA, 10% glycerol and 1% Triton^TM^ X-100. Lysate supernatants were incubated with each proteasome substrate for 30 min at 37 °C and measured by fluorescence (excitation, 355 nm; emission, 460 nm) by Ensipire^TM^ (PerkinElmer, Waltham, MA, USA).

### Statistical analysis

All experiments were performed in three independent replicates, and representative results were shown. Statistical analyses were performed by one-way ANOVA, followed by Tukey’s test. *P*-values < 0.05 were considered statistically significant.

## Additional Information

**How to cite this article**: Santoh, M. *et al.* Acetaminophen induces accumulation of functional rat CYP3A via polyubiquitination dysfunction. *Sci. Rep.* **6**, 21373; doi: 10.1038/srep21373 (2016).

## Supplementary Material

Supplementary Information
