# Supplementary material for: Acetaminophen induces accumulation of functional rat CYP3A via polyubiquitination dysfunction
Source: Sci Rep. 2016 Feb 22;6:21373. doi: 10.1038/srep21373 (PMC4761967; doi:10.1038/srep21373)
Supplement: Supplementary Information [file srep21373-s1.pdf]

Supplementary Information

Acetaminophen induces accumulation of functional rat CYP3A via polyubiquitination dysfunction

Masataka Santoh[1], Seigo Sanoh[1,*], Masashi Takagi[1], Yoko Ejiri[2], Yaichiro Kotake[1], and Shigeru Ohta[1]

[1]Graduate School of Biomedical and Health Sciences, Hiroshima University, 1-2-3 Kasumi, Minami-ku, Hiroshima, 734-8553, Japan, [2]Molding Component Business Department, New business Development Division, Kuraray Co., Ltd. 1-1-3 Otemachi, Chiyoda-ku, Tokyo, 100-8115, Japan.

[*]Corresponding author. E-mail address: sanoh@hiroshima-u.ac.jp

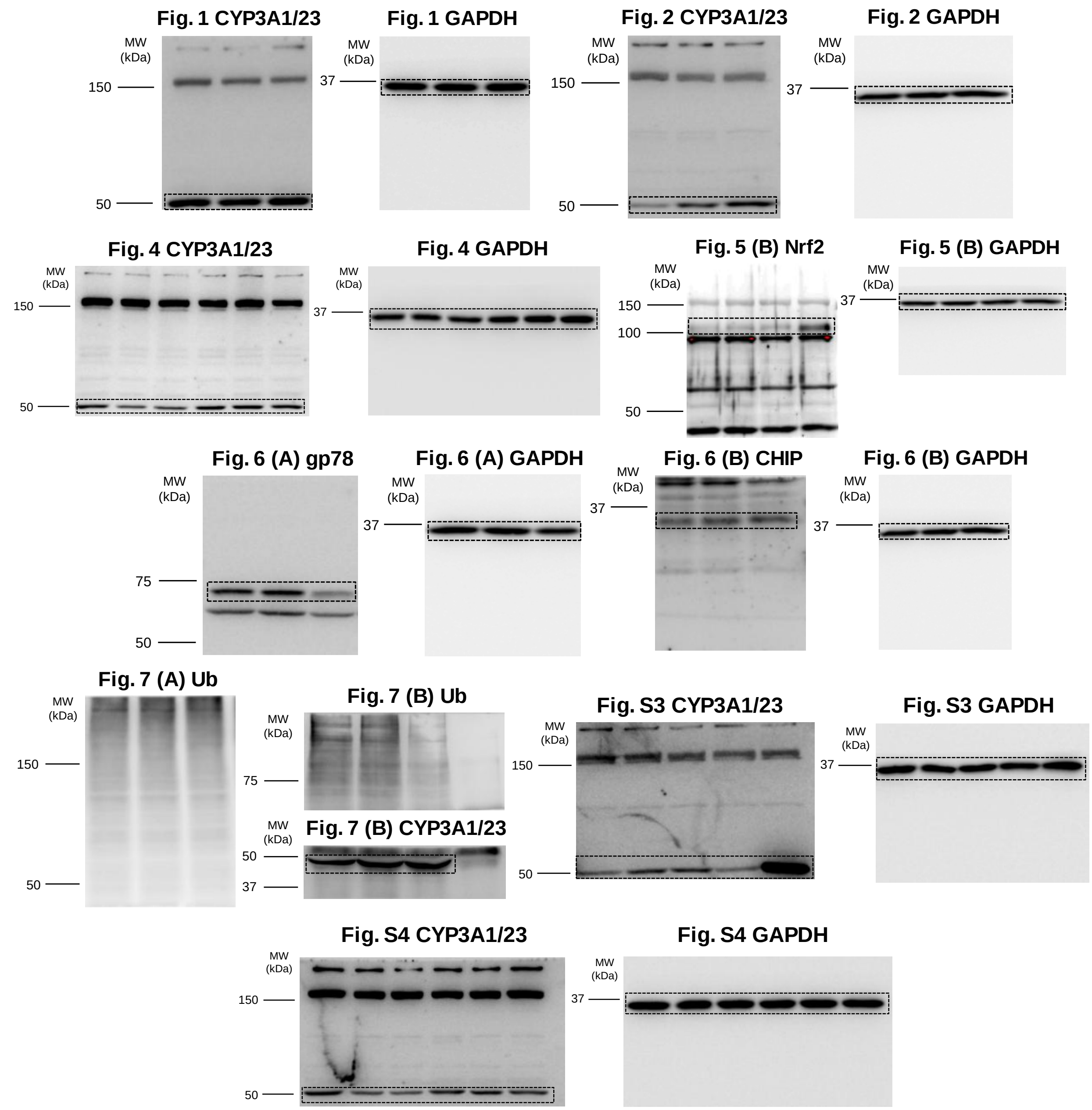

Figure S1. Images of full-length blots. Dotted lines show cropped images used in the figures.

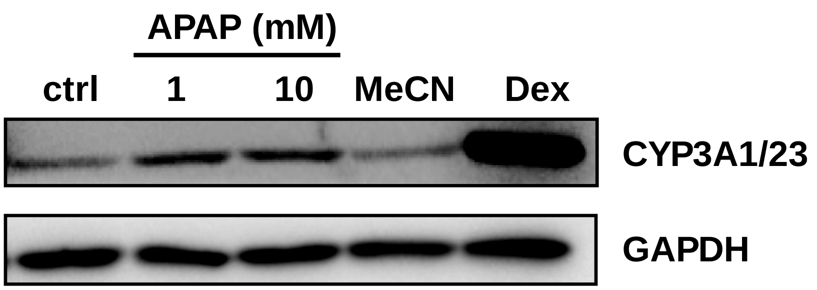

Figure S2. Evaluation of specificity of anti-CYP3A1 IgG using cell lysates form Dex-induced rat hepatocyte spheroids. Rat hepatocyte spheroids (day 5) were exposed to APAP (1 and 10 mM) and Dex (20 μM) for 24 h. The results of CYP3A1/23 and GAPDH immunoblotting analyses of cell lysates (5 μg of protein) are shown in the top panel. GAPDH was measured as a loading control. Cropped blots were shown and the full-length blots were presented in Supplementary Fig. 1.

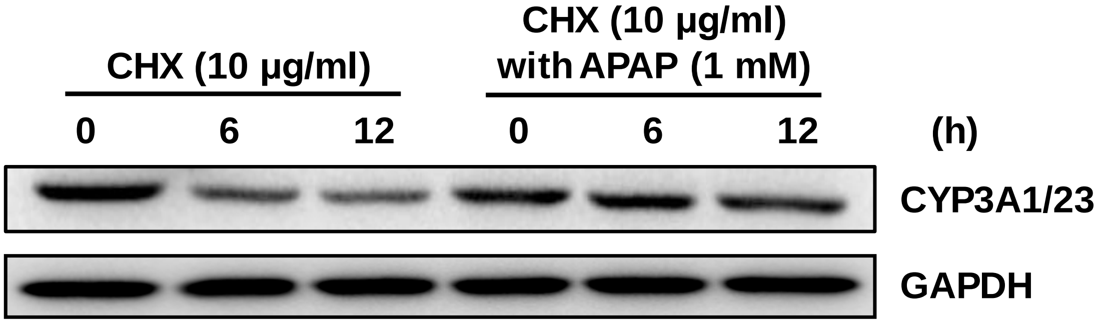

Figure S3. Effects of APAP (1 mM) on CYP3A1/23 protein degradation. Rat hepatocyte spheroids (day 5) were exposed to cycloheximide (10 µg/ml) with or without APAP (1 mM) for 6 or 12 h before harvesting. The results of CYP3A1/23 and GAPDH immunoblotting analyses of cell lysates (5 µg of protein) are shown. GAPDH was measured as a loading control. Cropped blots were shown and the full-length blots were presented in Supplementary Fig. 1.
